# Supplementary material for: Lactobacillus plantarum KAD protects against high-fat diet-induced hepatic complications in Swiss albino mice: Role of inflammation and gut integrity
Source: PLoS One. 2024 Nov 12;19(11):e0313548. doi: 10.1371/journal.pone.0313548 (PMC11556687; doi:10.1371/journal.pone.0313548)
Supplement: S1 File — (DOCX) [file pone.0313548.s001.docx]

**Supporting Information**

**S1 Table. Nutrient composition of experimental diets.**

| **Experimental diet** | **Content** | **Ingredients** | **Quantity** |
| --- | --- | --- | --- |
| **High-fat diet** | **1. Fat (45%)** | Lard | 215 gm |
|  |  | Commercial ghee | 100 gm |
|  | **2. Carbohydrate (43%)** | Sucrose | 200 gm |
|  |  | Wheat flour | 100 gm |
|  | **3. Protein (12 %)** | Casein powder | 85 gm |
|  | **Total** | | 700 gm |
|  | **4. Additional amino acids** | L- Cysteine (3 gm/Kg of total prepared feed) | 2.1 gm |
|  |  | L- Methionine (1.6 gm/Kg of total prepared feed) | 1.12 gm |
|  | **5. Vitamin-mineral mix** | L- Methionine (10 gm/Kg of total prepared feed) | 7 gm |
| **Normal pellet-based diet** | **1. Carbohydrate** | Wheat flour | 22.5 gm |
|  |  | Roasted bengal gram flour | 60 gm |
|  | **2. Protein** | Skim milk powder | 5 gm |
|  |  | Casein | 4 gm |
|  | **3. Fat** | Refined oil | 4 gm |
|  | **4. Salt mixture** | | 4 gm |
|  | **5. Vitamin mixture** | | 0.5 gm |
|  | **Total** | | 100 gm |

**S2 Table. Effects of dietary supplementation of *L. plantarum* KAD on different parameters among the experimental groups.** ND: Normal pellet diet fed normal control group; HFD: High-fat diet negative control group; LP: High-fat diet fed along with prophylactic *L. plantarum* KAD supplemented group

| **Test/ Group/ Value no.** | | **1** | **2** | **3** | **4** | **5** | **6** | **Mean**$\boldsymbol{\pm}$ **SD** |
| --- | --- | --- | --- | --- | --- | --- | --- | --- |
| **1. % Body weight gain** | **ND** | 33.33333333 | 31.81818182 | 36.36363636 | 38.0952381 | 36.84210526 | 35.29411765 | 35.29$\pm$2.34 |
|  | **HFD** | 91.66666667 | 91.30434783 | 95.23809524 | 95 | 94.73684211 | 94.73684211 | 93.78$\pm$1.79 |
|  | **LP** | 40 | 38.0952381 | 36.36363636 | 38.88888889 | 36.84210526 | 35 | 37.53$\pm$1.82 |
| **2. Liver Co-efficient** | **ND** | 1.535714286 | 1.413793103 | 1.633333333 | 1.448275862 | 1.576923077 | 1.652173913 | 1.54$\pm$0.10 |
|  | **HFD** | 5.239130435 | 5.227272727 | 5.585365854 | 5.051282051 | 5.621621622 | 5.72972973 | 5.41$\pm$0.27 |
|  | **LP** | 1.714285714 | 1.793103448 | 1.766666667 | 1.76 | 1.807692308 | 1.703703704 | 1.76$\pm$0.04 |
| **3. pgWAT Co-efficient** | **ND** | 1.142857143 | 1.137931034 | 1.2 | 1.206896552 | 1.346153846 | 1.47826087 | 1.25$\pm$0.13 |
|  | **HFD** | 3.934782609 | 4.090909091 | 3.951219512 | 3.717948718 | 3.135135135 | 3.216216216 | 3.67$\pm$0.41 |
|  | **LP** | 1.392857143 | 1.413793103 | 1.466666667 | 1.88 | 1.307692308 | 1.518518519 | 1.50$\pm$0.20 |
| **4. Intestine Co-efficient** | **ND** | 1.178571429 | 1.068965517 | 1.133333333 | 1.24137931 | 1.538461538 | 1.434782609 | 1.27$\pm$0.18 |
|  | **HFD** | 1.956521739 | 1.977272727 | 2.024390244 | 2.025641026 | 2.108108108 | 2.135135135 | 2.04$\pm$0.07 |
|  | **LP** | 1.5 | 1.310344828 | 1.333333333 | 1.48 | 1.384615385 | 1.296296296 | 1.38$\pm$0.09 |
| **5. AUC_glucose_ (mg h/dL)** | **ND** | 167 | 168.5 | 245.5 | 226.5 | 217.5 | 239 | 210.67$\pm$34.64 |
|  | **HFD** | 489.25 | 451.75 | 347 | 426.25 | 385 | 390.75 | 415.00$\pm$51.19 |
|  | **LP** | 176.75 | 212 | 243.75 | 194.5 | 197.25 | 212 | 206.04$\pm$22.63 |
| **6. Serum Insulin level (mU/l)** | **ND** | 3.390727432 | 3.418294322 | 3.363160543 | 3.280459874 | 3.308026763 | 3.335593653 | 3.35$\pm$0.05 |
|  | **HFD** | 9.124640488 | 7.19495821 | 6.919289313 | 8.021964901 | 8.297633798 | 7.470627107 | 7.84$\pm$0.81 |
|  | **LP** | 5.265275931 | 4.989607034 | 4.989607034 | 5.540944828 | 5.816613725 | 6.092282622 | 5.45$\pm$0.45 |
| **7. GHb %** | **ND** | 7.842579577 | 9.696916638 | 4.494091973 | 6.752095872 | 8.384103671 | 3.694773542 | 6.81$\pm$2.32 |
|  | **HFD** | 19.88114186 | 15.51787462 | 12.26623779 | 16.5041098 | 20.5390643 | 15.43103406 | 16.69$\pm$3.08 |
|  | **LP** | 9.428714346 | 9.536918545 | 6.54668132 | 4.623236539 | 8.950310086 | 8.884492107 | 8.00$\pm$1.98 |
| **8. HOMA-IR** | **ND** | 0.627284575 | 0.775724925 | 0.862762785 | 0.744445694 | 0.726222142 | 0.781640779 | 0.75$\pm$0.08 |
|  | **HFD** | 4.456474415 | 3.496270026 | 2.730812849 | 3.284727228 | 3.254331975 | 3.298530889 | 3.42$\pm$0.57 |
|  | **LP** | 0.909137644 | 0.923077301 | 1.230769735 | 0.956736474 | 1.147811775 | 1.803315656 | 1.16$\pm$0.34 |
| **9. Serum triglyceride level (mg/dL)** | **ND** | 90.78947368 | 98.68421053 | 97.36842105 | 102.6315789 | 77.89473684 | 93.42105263 | 93.46$\pm$8.67 |
|  | **HFD** | 221.0526316 | 217.1052632 | 200 | 201.3157895 | 213.1578947 | 221.0526316 | 212.28$\pm$9.48 |
|  | **LP** | 121.7105263 | 114.2105263 | 90.65789474 | 111.8421053 | 125 | 84.21052632 | 107.94$\pm$16.72 |
| **10. Srum cholesterol level (mg/dL)** | **ND** | 115.0776053 | 123.059867 | 75.16629712 | 89.80044346 | 73.17073171 | 72.50554324 | 91.46$\pm$22.44 |
|  | **HFD** | 240.1330377 | 222.8381375 | 199.556541 | 206.2084257 | 232.1507761 | 215.5210643 | 219.40$\pm$15.41 |
|  | **LP** | 125.7206208 | 95.12195122 | 87.80487805 | 109.7560976 | 77.16186253 | 109.7560976 | 100.89$\pm$17.56 |
| **11. Serum HDL-C level (mg/dL)** | **ND** | 52.67326733 | 60.1980198 | 45.14851485 | 52.67326733 | 35.74257426 | 31.98019802 | 46.40$\pm$10.88 |
|  | **HFD** | 10.34653465 | 11.85148515 | 10.53465347 | 13.92079208 | 20.69306931 | 16.36633663 | 13.95$\pm$4.01 |
|  | **LP** | 31.98019802 | 39.5049505 | 39.5049505 | 43.26732673 | 31.98019802 | 35.74257426 | 37.00$\pm$4.56 |
| **12. Serum LDL-C level (mg/dL)** | **ND** | 44.24644326 | 43.12500506 | 10.54409806 | 16.60086034 | 21.84921008 | 21.84113469 | 26.37$\pm$14.05 |
|  | **HFD** | 185.5759767 | 167.5655997 | 149.0218876 | 152.0244757 | 168.8261278 | 154.9442014 | 162.99$\pm$13.75 |
|  | **LP** | 69.39831756 | 32.77489546 | 30.16834861 | 44.12034978 | 20.18166451 | 57.17141804 | 42.30$\pm$18.36 |
| **13. Serum** **VLDL-C level (mg/dL)** | **ND** | 18.15789474 | 19.73684211 | 19.47368421 | 20.52631579 | 15.57894737 | 18.68421053 | 18.69$\pm$1.73 |
|  | **HFD** | 44.21052632 | 43.42105263 | 40 | 40.26315789 | 42.63157895 | 44.21052632 | 42.46$\pm$1.90 |
|  | **LP** | 24.34210526 | 22.84210526 | 18.13157895 | 22.36842105 | 25 | 16.84210526 | 21.59$\pm$3.34 |
| **14. Mean Adipocyte Area (μm^2^)** | **ND** | 1569.202519 | 2440.704416 | 1970.35785 | 2813.767267 | 1114.529184 | 1290.777463 | 1866.56$\pm$2717.95 |
|  | **HFD** | 20913.00319 | 14964.71005 | 13665.40843 | 18150.96123 | 14722.91246 | 15344.26634 | 16293.54$\pm$2714.05 |
|  | **LP** | 11304 | 7798.275487 | 4706.392847 | 6433.609507 | 4942.691663 | 3944.932799 | 6521.65$\pm$2714.05 |
| **15. Serum GGT level (U/L)** | **ND** | 5.211 | 2.895 | 4.632 | 5.79 | 4.053 | 2.316 | 4.15$\pm$1.34 |
|  | **HFD** | 12.159 | 10.422 | 9.843 | 12.159 | 11.58 | 11.001 | 11.19$\pm$0.95 |
|  | **LP** | 9.264 | 7.527 | 6.948 | 7.527 | 7.527 | 5.79 | 7.43$\pm$1.12 |
| **16. Serum ALT level (U/L)** | **ND** | 95.51733333 | 69.496 | 66.00533333 | 70.448 | 61.56266667 | 63.784 | 71.14$\pm$12.41 |
|  | **HFD** | 125.0293333 | 108.528 | 85.99733333 | 142.8 | 95.83466667 | 139.944 | 116.36$\pm$23.39 |
|  | **LP** | 97.73866667 | 91.392 | 73.304 | 97.73866667 | 71.71733333 | 72.03466667 | 83.99$\pm$12.97 |
| **17. Serum AST level (U/L)** | **ND** | 114.24 | 100.5946667 | 91.07466667 | 99.96 | 94.56533333 | 115.8266667 | 102.71$\pm$10.19 |
|  | **HFD** | 200.872 | 267.512 | 193.5733333 | 185.9573333 | 218.96 | 214.2 | 213.51$\pm$29.20 |
|  | **LP** | 120.904 | 98.37333333 | 89.80533333 | 130.1066667 | 105.9893333 | 98.056 | 107.21$\pm$15.35 |
| **18. Hepatic Triglyceride level(mg/g)** | **ND** | 2.578512397 | 3.019283747 | 3.033976125 | 4.789715335 | 4.958677686 | 4.532598714 | 3.82$\pm$1.05 |
|  | **HFD** | 21.20844812 | 21.4214876 | 18.28466483 | 20.97337006 | 19.61432507 | 18.90909091 | 20.07$\pm$1.32 |
|  | **LP** | 6.214876033 | 7.610651974 | 6.876033058 | 7.610651974 | 7.096418733 | 7.096418733 | 7.08$\pm$0.52 |
| **19. Hepatic SOD level (Units/mg tissue)** | **ND** | 0.165769912 | 0.177044248 | 0.175628319 | 0.16159292 | 0.169557522 | 0.164867257 | 0.17$\pm$0.01 |
|  | **HFD** | 0.061061947 | 0.064070796 | 0.058761062 | 0.048672566 | 0.047433628 | 0.056637168 | 0.06$\pm$0.01 |
|  | **LP** | 0.104424779 | 0.09840708 | 0.106902655 | 0.113451327 | 0.11539823 | 0.126548673 | 0.11$\pm$0.01 |
| **20. Hepatic** **Relative Concentration of GSH (%)** | **ND** | 85.87443946 | 84.32835821 | 96.84368737 | 96.44870349 | 96.62379421 | 90.51204819 | 91.77$\pm$5.71 |
|  | **HFD** | 39.42307692 | 54.34782609 | 41.66666667 | 28.40909091 | 23.17073171 | 35.71428571 | 37.12$\pm$10.91 |
|  | **LP** | 86.4806867 | 84.09090909 | 79.54545455 | 78.8590604 | 79.13907285 | 85.07109005 | 82.20$\pm$3.40 |
| **21. Hepatic MDA equivalent (nM/mg of tissue)** | **ND** | 0.023225806 | 0.036645161 | 0.022967742 | 0.023741935 | 0.034064516 | 0.027612903 | 0.12$\pm$0.01 |
|  | **HFD** | 0.121806452 | 0.138322581 | 0.102451613 | 0.11483871 | 0.13083871 | 0.120258065 | 0.04$\pm$0.01 |
|  | **LP** | 0.050064516 | 0.035741935 | 0.049806452 | 0.047741935 | 0.028645161 | 0.049806452 | 0.03$\pm$0.01 |
| **22.** **Hepatic** **Catalase level (Unit/g of tissue)** | **ND** | 8500 | 9272.727273 | 11333.33333 | 10200 | 10200 | 7846.153846 | 9558.70$\pm$1273.37 |
|  | **HFD** | 703.4482759 | 618.1818182 | 829.2682927 | 1478.26087 | 666.6666667 | 1009.90099 | 859.17$\pm$323.26 |
|  | **LP** | 6800 | 7846.153846 | 10200 | 7846.153846 | 7846.153846 | 7285.714286 | 7970.70$\pm$1170.99 |
| **23. Hepatic scoring** | **ND** | 0 | 2 | 0 | 0 | 1 | 1 | 0.67$\pm$0.82 |
|  | **HFD** | 6 | 5 | 5 | 5 | 4 | 5 | 5.00$\pm$0.63 |
|  | **LP** | 2 | 2 | 2 | 2 | 2 | 1 | 1.83$\pm$0.41 |
| **24. Serum LBP level (ng/mL)** | **ND** | 205.5172414 | 271.7241379 | 185.5172414 | 109.6551724 | 182.7586207 | 224.137931 | 196.55$\pm$53.58 |
|  | **HFD** | 1011.724138 | 1081.37931 | 1069.655172 | 1041.37931 | 970.3448276 | 1083.448276 | 1042.99$\pm$44.90 |
|  | **LP** | 246.8965517 | 324.8275862 | 327.5862069 | 337.9310345 | 264.8275862 | 220 | 287.01$\pm$49.52 |
| **25. Colonic SOD level (Units/mg tissue)** | **ND** | 0.096697248 | 0.096146789 | 0.108073394 | 0.102201835 | 0.1 | 0.097431193 | 0.1$\pm$0.005 |
|  | **HFD** | 0.01706422 | 0.017981651 | 0.016146789 | 0.015412844 | 0.017431193 | 0.017247706 | 0.017$\pm$0.001 |
|  | **LP** | 0.069541284 | 0.068256881 | 0.07266055 | 0.07559633 | 0.070275229 | 0.076880734 | 0.072$\pm$0.003 |
| **26. Colonic Relative Concentration of GSH (%)** | **ND** | 74.3220339 | 74.95867769 | 77.21804511 | 77.55555556 | 76.14173228 | 76.51162791 | 76.12$\pm$1.27 |
|  | **HFD** | 24.06015038 | 24.81389578 | 21.90721649 | 26.27737226 | 25.91687042 | 24.06015038 | 24.51$\pm$1.57 |
|  | **LP** | 62.21945137 | 72.125115 | 62.077597 | 63.71257485 | 69.01840491 | 73.06666667 | 67.04$\pm$5.00 |
| **27.Colonic MDA equivalent (nM/mg of tissue)** | **ND** | 0.035096774 | 0.035096774 | 0.032 | 0.023225806 | 0.035870968 | 0.032 | 0.032$\pm$0.005 |
|  | **HFD** | 0.12 | 0.131612903 | 0.118967742 | 0.127741935 | 0.132387097 | 0.13883871 | 0.128$\pm$0.008 |
|  | **LP** | 0.054709677 | 0.057548387 | 0.047483871 | 0.054193548 | 0.050580645 | 0.047483871 | 0.052$\pm$0.004 |
| **28. Colonic** **Catalase level (Unit/g of tissue)** | **ND** | 3187.5 | 2833.333333 | 3187.5 | 3000 | 2684.210526 | 3000 | 2982.09$\pm$197.93 |
|  | **HFD** | 435.8974359 | 435.8974359 | 404.7619048 | 395.3488372 | 425 | 414.6341463 | 418.59$\pm$16.66 |
|  | **LP** | 1214.285714 | 1645.16129 | 1416.666667 | 927.2727273 | 944.4444444 | 980.7692308 | 1188.10$\pm$294.01 |
| **29. Macroscopic score** | **ND** | 1 | 1 | 1 | 0 | 1 | 2 | 1.00$\pm$0.63 |
|  | **HFD** | 8 | 7 | 5 | 5 | 3 | 1 | 4.83$\pm$2.56 |
|  | **LP** | 4 | 2 | 3 | 3 | 1 | 2 | 2.50$\pm$1.05 |
| **30. Microscopic score** | **ND** | 2 | 2 | 1 | 1 | 1 | 0 | 1.17$\pm$0.75 |
|  | **HFD** | 4 | 5 | 5 | 5 | 7 | 3 | 4.83$\pm$1.33 |
|  | **LP** | 2 | 2 | 3 | 3 | 4 | 2 | 2.67$\pm$0.82 |
| **31. Serum CRP level (mg/L)** | **ND** | 1.431576145 | 1.341054313 | 1.383652822 | 1.404952077 | 1.122736954 | 1.466187433 | 1.36$\pm$0.12 |
|  | **HFD** | 1.817625133 | 1.977369542 | 1.905484558 | 2.097177849 | 2.129126731 | 1.897497338 | 1.97$\pm$0.12 |
|  | **LP** | 1.572683706 | 1.591320554 | 1.506123536 | 1.535410011 | 1.516773163 | 1.522097977 | 1.54$\pm$0.03 |
| **32. Serum TNF-α level (pg/mL)** | **ND** | 35.54545455 | 34.03030303 | 23.12121212 | 26.45454545 | 27.36363636 | 31 | 29.59±4.77 |
|  | **HFD** | 147.3636364 | 141 | 144.3333333 | 148.5757576 | 144.6363636 | 147.969697 | 145.65±2.87 |
|  | **LP** | 64.03030303 | 67.66666667 | 63.72727273 | 58.57575758 | 57.66666667 | 53.42424242 | 60.85±5.20 |
| **33. Serum IL-6 level (pg/mL)** | **ND** | 14.2 | 19.7 | 18.2 | 15.7 | 20.7 | 15.2 | 17.28±2.63 |
|  | **HFD** | 39.2 | 38.7 | 37.7 | 42.2 | 41.7 | 41.2 | 40.12±1.83 |
|  | **LP** | 23.2 | 27.2 | 26.7 | 27.7 | 24.7 | 25.2 | 25.78±1.72 |
| **34. Hepatic TNF-α level (pg/mg tissue)** | **ND** | 3.378787879 | 7.621212121 | 6.106060606 | 6.409090909 | 5.348484848 | 4.893939394 | 5.63±1.45 |
|  | **HFD** | 33.53030303 | 34.43939394 | 32.77272727 | 32.16666667 | 29.74242424 | 28.37878788 | 31.84±2.32 |
|  | **LP** | 8.378787879 | 8.833333333 | 9.136363636 | 7.924242424 | 7.621212121 | 6.863636364 | 8.13±0.83 |
| **35. Hepatic IL-6 level (pg/mg tissue)** | **ND** | 10.6 | 17.1 | 10.35 | 13.1 | 16.85 | 13.35 | 13.56±2.34 |
|  | **HFD** | 32.85 | 38.35 | 40.35 | 41.85 | 39.6 | 38.1 | 38.52±3.10 |
|  | **LP** | 19.35 | 25.35 | 22.1 | 29.6 | 20.85 | 22.1 | 23.23±3.70 |
| **36. Colonic TNF-α level (pg/mg tissue)** | **ND** | 8.53030303 | 10.34848485 | 11.71212121 | 13.53030303 | 13.53030303 | 13.98484848 | 11.94±2.17 |
|  | **HFD** | 60.8030303 | 59.28787879 | 57.77272727 | 58.68181818 | 60.04545455 | 59.59090909 | 59.36±1.06 |
|  | **LP** | 27.92424242 | 28.37878788 | 29.13636364 | 24.74242424 | 23.68181818 | 22.16666667 | 26.01±2.86 |
| **37. Colonic IL-6 level (pg/mg tissue)** | **ND** | 20.1 | 20.6 | 23.1 | 24.1 | 22.1 | 17.6 | 21.27±2.34 |
|  | **HFD** | 103.6 | 109.35 | 108.85 | 108.1 | 105.85 | 110.6 | 107.73±2.56 |
|  | **LP** | 31.6 | 34.1 | 35.6 | 34.85 | 35.85 | 33.1 | 34.18±1.62 |
| **38. Adipose tissue TNF-α (pg/mg tissue)** | **ND** | 0.454545455 | 1.96969697 | 3.484848485 | 3.484848485 | 1.96969697 | 0.454545455 | 1.97±1.36 |
|  | **HFD** | 1.96969697 | 5 | 3.484848485 | 3.484848485 | 1.96969697 | 5 | 3.48$\pm$1.36 |
|  | **LP** | 3.484848485 | 3.484848485 | 5 | 0.454545455 | 1.96969697 | 0.454545455 | 2.47$\pm$1.83 |
| **39. Adipose tissue IL-6 (pg/mg tissue)** | **ND** | 213.5 | 193.5 | 183.5 | 193.5 | 188.5 | 221 | 198.92±14.87 |
|  | **HFD** | 483.5 | 453.5 | 488.5 | 421 | 458.5 | 471 | 462.67±24.53 |
|  | **LP** | 241 | 236 | 243.5 | 248.5 | 276 | 273.5 | 253.08±17.28 |

**S3 Table.** **Effects of dietary supplementation of *L. plantarum* KAD on hepatic scoring of different experimental groups.** ND: Normal pellet diet fed normal control group; HFD: High-fat diet negative control group; LP: High-fat diet fed along with prophylactic *L. plantarum* KAD supplemented group

The criteria and the scoring patterns are as follows:

| **Parameters** | **Scoring Criteria** | **Score** |
| --- | --- | --- |
| A. Steatosis | <5% | 0 |
|  | 5-33% | 1 |
|  | >33-66% | 2 |
|  | >66% | 3 |
| B. Inflammation | Absent | 0 |
|  | Rare | 1 |
|  | Moderate | 2 |
|  | Severe | 3 |
| C. Hepatocyte ballooning | Absent | 0 |
|  | Few balloon cells | 1 |
|  | Many | 2 |

Based on the above criteria, the obtained scores are as follows:

| **Experimental**  **individuals** | **A** | **B** | **C** | **Score** | **Mean**$\boldsymbol{\pm}$ **SD** |
| --- | --- | --- | --- | --- | --- |
| **ND1** | 0 | 1 | 0 | 0 | 0.67$\pm$0.82 |
| **ND2** | 0 | 1 | 1 | 2 |  |
| **ND3** | 0 | 0 | 0 | 0 |  |
| **ND4** | 0 | 0 | 0 | 0 |  |
| **ND5** | 0 | 1 | 0 | 1 |  |
| **ND6** | 0 | 1 | 0 | 1 |  |
| **HFD1** | 2 | 2 | 2 | 6 | 5.00$\pm$0.63 |
| **HFD2** | 1 | 3 | 1 | 5 |  |
| **HFD3** | 1 | 2 | 2 | 5 |  |
| **HFD4** | 1 | 3 | 1 | 5 |  |
| **HFD5** | 2 | 1 | 1 | 4 |  |
| **HFD6** | 1 | 2 | 2 | 5 |  |
| **LP1** | 0 | 1 | 1 | 2 | 1.83$\pm$0.41 |
| **LP2** | 1 | 1 | 0 | 2 |  |
| **LP3** | 1 | 1 | 0 | 2 |  |
| **LP4** | 1 | 0 | 1 | 2 |  |
| **LP5** | 0 | 1 | 1 | 2 |  |
| **LP6** | 0 | 1 | 0 | 1 |  |

**S4 Table.** **Effects of dietary supplementation of *L. plantarum* KAD on macroscopic scoring of colonic integrity among different experimental groups.** ND: Normal pellet diet fed normal control group; HFD: High-fat diet negative control group; LP: High-fat diet fed along with prophylactic *L. plantarum* KAD supplemented group

The criteria and the scoring patterns are as follows:

| **Parameters** | **Scoring Criteria** | **Score** |
| --- | --- | --- |
| A. Diarrhoea | Present | 1 |
|  | Absent | 0 |
| B. Colonic adhesion to other internal organs | No adhesion | 0 |
|  | Minor adhesion (Colon can be easily separated) | 1 |
|  | Major adhesion (Colonic tissues juxtaposed with other connective tissues of internal organs) | 2 |
| C. Presence of inflammatory ulcerations | No ulceration | 0 |
|  | Focal hyperemia without prominent ulceration | 1 |
|  | Inflammatory ulcerative changes at less than 2 sites | 2 |
|  | Inflammatory ulcerative changes at more than 2 sites | 3 |
| D. Colonic Length | 11-13 cm | 0 |
|  | 9-11 cm | 1 |
|  | 7-9 cm | 2 |
|  | 5-7 cm | 3 |

Based on the above criteria, the obtained scores are as follows:

| **Experimental**  **Individuals*** | **A** | **B** | **C** | **D** | **Score** | **Mean**$\boldsymbol{\pm}$ **SD** |
| --- | --- | --- | --- | --- | --- | --- |
| **ND1** | 0 | 0 | 0 | 1 | 1 | 1.00$\pm$0.63 |
| **ND2** | 0 | 0 | 0 | 1 | 1 |  |
| **ND3** | 0 | 0 | 0 | 1 | 1 |  |
| **ND4** | 0 | 0 | 0 | 0 | 0 |  |
| **ND5** | 0 | 0 | 1 | 0 | 1 |  |
| **ND6** | 0 | 1 | 1 | 0 | 2 |  |
| **HFD1** | 1 | 2 | 3 | 2 | 8 | 4.83$\pm$2.56 |
| **HFD2** | 1 | 2 | 2 | 2 | 7 |  |
| **HFD3** | 1 | 1 | 2 | 1 | 5 |  |
| **HFD4** | 0 | 1 | 1 | 3 | 5 |  |
| **HFD5** | 0 | 0 | 3 | 0 | 3 |  |
| **HFD6** | 0 | 0 | 0 | 1 | 1 |  |
| **LP1** | 1 | 1 | 1 | 1 | 4 | 2.50$\pm$1.05 |
| **LP2** | 0 | 0 | 1 | 1 | 2 |  |
| **LP3** | 0 | 1 | 1 | 1 | 3 |  |
| **LP4** | 0 | 0 | 2 | 1 | 3 |  |
| **LP5** | 0 | 1 | 0 | 0 | 1 |  |
| **LP6** | 0 | 2 | 0 | 0 | 2 |  |

**S5 Table.** **Effects of dietary supplementation of *L. plantarum* KAD on microscopic scoring of colonic integrity among different experimental groups.** ND: Normal pellet diet fed normal control group; HFD: High-fat diet negative control group; LP: High-fat diet fed along with prophylactic *L. plantarum* KAD supplemented group

The criteria and the scoring patterns are as follows:

| **Parameters** | **Scoring Criteria** | **Score** |
| --- | --- | --- |
| A. Loss of muscle architecture indicated by muscle thickness | All 3 layers are intact | 0 |
|  | Partial reduction in thickness | 1 |
|  | Moderate reduction in thickness | 2 |
|  | Severe reduction in thickness | 3 |
| B. Immune Cell infiltration | Absent | 0 |
|  | Slight | 1 |
|  | Moderate | 2 |
|  | Severe | 3 |
| C. Crypt abscesses formation | Absent | 0 |
|  | Present | 1 |
| D. Goblet cell depletion | Absent | 0 |
|  | Present | 1 |

Based on the above criteria, the obtained scores are as follows:

| **Experimental**  **individuals** | **A** | **B** | **C** | **D** | **Score** | **Mean**$\boldsymbol{\pm}$ **SD** |
| --- | --- | --- | --- | --- | --- | --- |
| **ND1** | 0 | 1 | 0 | 1 | 2 | 1.17$\pm$0.75 |
| **ND2** | 0 | 1 | 0 | 1 | 2 |  |
| **ND3** | 0 | 0 | 0 | 1 | 1 |  |
| **ND4** | 0 | 0 | 1 | 0 | 1 |  |
| **ND5** | 1 | 0 | 0 | 0 | 1 |  |
| **ND6** | 0 | 0 | 0 | 0 | 0 |  |
| **HFD1** | 2 | 1 | 0 | 1 | 4 | 4.83$\pm$1.33 |
| **HFD2** | 3 | 1 | 0 | 1 | 5 |  |
| **HFD3** | 1 | 2 | 1 | 1 | 5 |  |
| **HFD4** | 1 | 2 | 1 | 1 | 5 |  |
| **HFD5** | 2 | 3 | 1 | 1 | 7 |  |
| **HFD6** | 1 | 1 | 1 | 0 | 3 |  |
| **LP1** | 0 | 1 | 0 | 1 | 2 | 2.67$\pm$0.82 |
| **LP2** | 1 | 1 | 0 | 0 | 2 |  |
| **LP3** | 2 | 0 | 0 | 1 | 3 |  |
| **LP4** | 2 | 0 | 1 | 0 | 3 |  |
| **LP5** | 2 | 1 | 1 | 0 | 4 |  |
| **LP6** | 0 | 2 | 0 | 0 | 2 |  |

**S6 Table. 16s V3-V4 fecal metagenomic analysis of different experimental groups. Data are shown in terms of relative abundance.** ND: Normal pellet diet fed normal control group; HFD: High-fat diet negative control group; LP: High-fat diet fed along with prophylactic *L. plantarum* KAD supplemented group

| **Taxon/ Experimental groups** | | **Relative abundance (%)** | | |
| --- | --- | --- | --- | --- |
|  |  | **ND** | **HFD** | **LP** |
| **Phylum** | Actinobacteria | 0.90 | 7.44 | 10.05 |
|  | Bacteroidetes | 51.61 | 66.20 | 56.08 |
|  | Firmicutes | 38.73 | 20.46 | 27.57 |
|  | Proteobacteria | 5.16 | 5.82 | 2.63 |
|  | Spirochaetes | 2.98 | 0.02 | 3.51 |
| **Family** | Aeromonadaceae | 2.05 | 0.52 | 0.19 |
|  | Atopobiaceae | 0.01 | 2.80 | 0.00 |
|  | Bacteroidaceae | 24.72 | 11.04 | 5.91 |
|  | Bifidobacteriaceae | 0.74 | 3.87 | 9.71 |
|  | Blattabacteriaceae | 0.06 | 3.07 | 1.13 |
|  | Desulfovibrionaceae | 0.57 | 1.54 | 0.23 |
|  | Enterobacteriaceae | 0.03 | 2.68 | 0.08 |
|  | Helicobacteraceae | 2.14 | 0.62 | 0.96 |
|  | Lachnospiraceae | 9.94 | 0.54 | 2.42 |
|  | Lactobacillaceae | 13.65 | 0.08 | 5.88 |
|  | Muribaculaceae | 0.25 | 4.39 | 1.63 |
|  | Oscillospiraceae | 0.56 | 1.04 | 0.49 |
|  | Prevotellaceae | 16.55 | 11.44 | 25.26 |
|  | Rikenellaceae | 0.34 | 1.97 | 2.08 |
|  | Spirochaetaceae | 2.98 | 0.02 | 3.51 |
|  | Staphylococcaceae | 0.15 | 0.60 | 4.34 |
|  | Tannerellaceae | 0.39 | 0.62 | 1.46 |
|  | Unclassified Bacteroidales | 8.78 | 32.87 | 17.87 |
|  | Unclassified Clostridiales | 13.56 | 11.61 | 12.82 |
| **Genus** | *Acetobacter* | 0.00 | 0.00 | 0.78 |
|  | *Aeromonas* | 2.05 | 0.52 | 0.19 |
|  | *Alistipes* | 0.28 | 1.76 | 1.85 |
|  | *Bacteroides* | 24.72 | 11.04 | 5.91 |
|  | *Bifidobacterium* | 0.74 | 3.87 | 9.70 |
|  | *Blattabacterium* | 0.06 | 3.07 | 1.13 |
|  | *Blautia* | 8.48 | 0.04 | 0.05 |
|  | *Clostridium* | 0.03 | 5.40 | 0.14 |
|  | *Desulfovibrio* | 0.43 | 0.88 | 0.10 |
|  | *Helicobacter* | 2.14 | 0.62 | 0.96 |
|  | *Lactobacillus* | 13.65 | 0.08 | 5.69 |
|  | *Muribaculum* | 0.10 | 2.84 | 1.15 |
|  | *Olsenella* | 0.01 | 2.78 | 0.00 |
|  | *Prevotella* | 15.30 | 10.82 | 24.62 |
|  | *Staphylococcus* | 0.15 | 0.60 | 4.25 |
|  | *Tannerella* | 0.39 | 0.62 | 1.46 |
|  | *Treponema* | 2.84 | 0.01 | 3.39 |
|  | *Unclassified Bacteroidales* | 8.78 | 32.87 | 17.87 |
|  | *Unclassified Clostridiales* | 13.56 | 11.61 | 12.82 |
|  | *Unclassified Desulfovibrionaceae* | 0.14 | 0.67 | 0.13 |
|  | *Unclassified Enterobacteriaceae* | 0.03 | 2.67 | 0.07 |
|  | *Unclassified Flavobacteriales* | 0.48 | 0.17 | 0.14 |
|  | *Unclassified Lachnospiraceae* | 0.53 | 0.47 | 2.33 |
|  | *Unclassified Muribaculaceae* | 0.15 | 1.55 | 0.48 |
|  | *Unclassified Prevotellaceae* | 1.25 | 0.62 | 0.65 |
| **Species** | *Bacteroides sp.* | 4.92 | 9.22 | 5.28 |
|  | *Bacteroides vulgatus* | 17.03 | 0.19 | 0.29 |
|  | *Clostridioides difficile* | 0.04 | 0.42 | 0.01 |
|  | *Clostridium sp.* | 0.03 | 0.15 | 0.03 |
|  | *Clostridium tyrobutyricum* | 0.00 | 5.24 | 0.11 |
|  | *Desulfovibrio fairfieldensis* | 0.35 | 0.52 | 0.04 |
|  | *Desulfovibrio sp.* | 0.08 | 0.35 | 0.05 |
|  | *Eggerthella sp.* | 0.01 | 0.16 | 0.01 |
|  | *Flavobacterium sp.* CJ74 | 0.01 | 0.13 | 0.08 |
|  | *Lactobacillus backii* | 8.54 | 0.02 | 2.78 |
|  | *Lactobacillus brevis* | 0.06 | 0.00 | 0.06 |
