# Supplementary material for: Lactobacillus plantarum KAD protects against high-fat diet-induced hepatic complications in Swiss albino mice: Role of inflammation and gut integrity
Source: PLoS One. 2024 Nov 12;19(11):e0313548. doi: 10.1371/journal.pone.0313548 (PMC11556687; doi:10.1371/journal.pone.0313548)
Supplement: Attachment — Submitted filename: Response to Reviewers.docx [file pone.0313548.s002.docx]

**Answers to Reviewers’ Comments**

**Journal Name:**	PLOS ONE

**Manuscript Title:**	“*Lactobacillus plantarum* KAD protects against High-Fat Diet-Induced Hepatic Complications in Swiss Albino Mice: Role of Inflammation and Gut Integrity”

**Manuscript Number:** PONE-D-24-27060

Authors are sincerely thankful to the Editor and Reviewers for their time, constructive comments and suggestions for improvement of the manuscript and showing their interest on the research article.

**Editorial Comments:**

**Journal Requirements:**

When submitting your revision, we need you to address these additional requirements.

**1. Please ensure that your manuscript meets PLOS ONE's style requirements, including those for file naming. The PLOS ONE style templates can be found at**

<https://journals.plos.org/plosone/s/file?id=wjVg/PLOSOne_formatting_sample_main_body.pdf>

<https://journals.plos.org/plosone/s/file?id=ba62/PLOSOne_formatting_sample_title_authors_affiliations.pdf>

**Response:** Thank you for your valuable suggestion. Changes are made in the manuscript according to the Journal’s requirements.

**2. To comply with PLOS ONE submissions requirements, in your Methods section, please provide additional information regarding the experiments involving animals and ensure you have included details on (1) methods of sacrifice, (2) methods of anesthesia and/or analgesia, and (3) efforts to alleviate suffering.**

**Response:** Thank you for your kind feedback. Necessary changes are done in section 2.3 as per Journal’s requirements.

**3. Thank you for stating the following in your Competing Interests section: On behalf of all authors I disclose that there is no competing interests that could be perceived to bias this work.**

Please complete your Competing Interests on the online submission form to state any Competing Interests. If you have no competing interests, please state ""The authors have declared that no competing interests exist."", as detailed online in our guide for authors at <http://journals.plos.org/plosone/s/submit-now>

This information should be included in your cover letter; we will change the online submission form on your behalf.

**4. We note that your Data Availability Statement is currently as follows: All relevant data are within the manuscript and its Supporting Information files.**

Please confirm at this time whether or not your submission contains all raw data required to replicate the results of your study. Authors must share the “minimal data set” for their submission. PLOS defines the minimal data set to consist of the data required to replicate all study findings reported in the article, as well as related metadata and methods (<https://journals.plos.org/plosone/s/data-availability#loc-minimal-data-set-definition>).

**For example, authors should submit the following data:**

- The values behind the means, standard deviations and other measures reported;

- The values used to build graphs;

- The points extracted from images for analysis.

Authors do not need to submit their entire data set if only a portion of the data was used in the reported study.

If your submission does not contain these data, please either upload them as Supporting Information files or deposit them to a stable, public repository and provide us with the relevant URLs, DOIs, or accession numbers. For a list of recommended repositories, please see <https://journals.plos.org/plosone/s/recommended-repositories>.

If there are ethical or legal restrictions on sharing a de-identified data set, please explain them in detail (e.g., data contain potentially sensitive information, data are owned by a third-party organization, etc.) and who has imposed them (e.g., an ethics committee). Please also provide contact information for a data access committee, ethics committee, or other institutional body to which data requests may be sent. If data are owned by a third party, please indicate how others may request data access.

**Response:** S2, and S6 Tables are attached in the supporting information file in support of values behind the means, standard deviations and other measures reported.

**Additional Editor Comments:**

**This manuscript number (PONE-D-24-27060) is entitled "Oral supplementation of Lactobacillus plantarum KAD Prevents High-Fat Diet-Induced Hepatic Complications in Swiss Albino Mice: Role of Inflammation and Gut Integrity". The novelty of the manuscript is good although the analyses are not sufficient in this work. This article can be considered for publication after doing the following major revisions:**

**1. This paper is worthy of publication although the novelty should be addressed clearly in the abstract and introduction.**

**Response:** Thank you for your valuable suggestion. Necessary changes are made in the abstract section (line number: 23-27) and introduction section (line number: 87-89).

**2. The abstract is concise but could benefit from a clearer statement of the study's objectives, methods, main findings, and significance. Start by clearly stating the objective of the study. Briefly describe the methodology, emphasizing the study design and key experimental procedures. Highlight the most significant results, particularly those that demonstrate the impact of Lactobacillus plantarum KAD on hepatic complications. Conclude with a sentence on the study’s implications for future research or clinical practice.**

**Response:** Authors are thankful for your valuable feedback. All the changes are made in the abstract section accordingly.

**3. The introduction outlines the background and the need for the study but could be more focused on the research gap. Streamline the introduction by focusing on the specific gaps in current research that your study addresses. For instance, emphasize the novelty of exploring Lactobacillus plantarum KAD’s role in hepatic complications within the context of high-fat diet-induced obesity. You might also want to more explicitly state the hypothesis or primary research question.**

**Response:** Thank you for your valuable suggestion. Specific gaps of current research are mentioned in the introduction section (line no. 80-84). As per your suggestion, introduction is streamlined and hypothesis or primary research question is added at the last paragraph of introduction.

**4. The methods are detailed, but some sections might be too dense. Consider breaking down the methods into more specific subheadings for clarity, such as "Animal Grouping and Diet," "Biochemical Analysis," "Histological Examination," etc. This will make it easier for readers to follow your procedures. Additionally, ensure that all procedures, especially those involving statistical analyses, are described with enough detail to allow replication.**

**Response:** Authors are thankful to the Additional Editor for his/her esteemed suggestion. The method section has been updated accordingly with all the modifications.

**5. The results section is comprehensive but could benefit from more emphasis on the significance of the findings. Instead of just presenting the data, you can enhance the narrative by linking the findings back to the research questions. For instance, when discussing the lipid profile or histological findings, briefly state what these results imply about the effectiveness of Lactobacillus plantarum KAD. Use subheadings to organize the results logically, perhaps under themes like "Glycemic Control," "Lipid Metabolism," "Hepatic Function," etc.**

**Response:** Thank you for your suggestion. Result sections are changed as per your suggestions. For better understanding of the experimental findings section 3.4 and 3.5 are further categorized.

**6. The discussion provides a good analysis but can be more tightly linked to the results. Start by summarizing the main findings in a few sentences. Then, critically evaluate these findings in the context of existing literature, discussing how your study confirms, contradicts, or extends previous research. Ensure that the discussion logically follows from the results presented, addressing potential limitations of the study and suggesting areas for future research. Consider framing the discussion around how Lactobacillus plantarum KAD could be integrated into therapeutic strategies for metabolic disorders.**

**Response:** Summary of the experiment is added in the first paragraph of the discussion as per your valuable suggestion. We have changed the findings in the context of existing literature accordingly and added potential limitations of this study (line no. 435,436; 450-452; 467-470; 544-549). Necessary changes are made in the discussion section. Finally, discussion is framed around the therapeutic strategy of using L. plantarum KAD in the line no. 547-551.

**7. The conclusion is currently embedded within the discussion. Create a distinct conclusion section that succinctly restates the main findings, the potential clinical implications, and the next steps for research. This will help to underscore the importance of your work.**

**Response:** Authors are thankful to the Additional Editor for his/her valuable suggestion. Conclusion section is added in the manuscript ,and written as per your suggestion including necessary amends.

**8. The choice of inflammatory markers (CRP, TNF-α, IL-6) is appropriate but limited. Including additional markers, such as IL-10 or IL-1β, could provide a more comprehensive picture of the inflammatory response and further substantiate the claims made regarding anti-inflammatory effects.**

**Response:** Thank you for your insightful feedback regarding the inflammatory markers used in our study. We believe that the selection of CRP, TNF-α, and IL-6 is appropriate and sufficient for our research objectives, as these markers are well-established indicators of inflammation in diet-induced hepatic complications. Numerous studies have shown that elevated levels of these markers correlate with the pathogenesis of non-alcoholic fatty liver disease (NAFLD) and metabolic syndrome, making them reliable for assessing the anti-inflammatory effects of interventions like probiotic supplementation [1,2]. While we acknowledge that additional markers such as IL-10 or IL-1β could provide further insights, our focus on CRP, TNF-α, and IL-6 aligns with our goal of evaluating the protective effects of *Lactobacillus plantarum* KAD in a broad context. In future experiments, we plan to explore more detailed analyses, including metagenomics, and will consider incorporating additional inflammatory markers to enhance our understanding of the inflammatory response [3]. Thank you again for your valuable suggestions.

**9. While the study shows that Lactobacillus plantarum KAD has beneficial effects, the mechanistic pathways are not thoroughly explored. The discussion would benefit from more detailed hypotheses or evidence about how the probiotic strain exerts its effects, particularly in relation to gut microbiota modulation and hepatic function.**

**Response:**

**10. The discussion sometimes overstates the significance of the findings without sufficient qualification. For example, the claims about the potential therapeutic benefits of Lactobacillus plantarum KAD should be tempered by acknowledging the limitations of extrapolating animal model results to human health outcomes.**

**Response:** Thank you for your valuable suggestion. Discussion section is changed eliminating such overstatements accordingly.

**11. The figures and tables are informative but may need more detailed legends. Ensure that each figure and table have a comprehensive legend that explains all abbreviations and provides enough context for understanding without referring to the text. Consider whether the figures could be reordered or combined to tell a clearer story.**

**Response:** Thank you for your valuable advice. Figure legends are detailed as per your suggestion without changing the order.

**12. The section on gut microbiota analysis could be expanded to include more details on the bioinformatics methods used. For example, the criteria for sequence quality control, taxonomy assignment, and the statistical methods used to compare the microbiota compositions between groups should be clearly stated.**

**Response:** Thank you for your constructive feedback regarding the gut microbiota analysis section of our manuscript. We acknowledge the importance of providing detailed information on the bioinformatics methods employed in our study. However, expanding this section significantly may lead to unnecessary length, as our primary focus is on the protective effects of Lactobacillus plantarum KAD. Nevertheless, we would like to summarize the key bioinformatics methods used in our analysis.For sequence quality control, we utilized FASTQC and MultiQC to assess the raw sequence data, ensuring high-quality reads. Trimming of adapters and low-quality reads was performed using Trim Galore, followed by merging of paired-end reads and chimera removal. Operational Taxonomic Unit (OTU) abundance was calculated using QIIME and KRAKEN workflows, which are effective for accurate investigations at the genus level. Taxonomy assignment was based on databases such as SILVA, GREENGENES, and NCBI, with classification determined by percentage coverage and identity (Biokart India Pvt. Ltd., 2023). To compare microbiota compositions between groups, we implemented statistical analyses including alpha and beta diversity metrics, which help in understanding the diversity and composition of microbial communities. Features with identical values across all samples were excluded, as were those appearing in only one sample, which are considered artifacts. Additionally, we applied low count and low variance filters to enhance the robustness of our statistical analysis, ensuring that only informative features were retained for downstream analysis (Biokart India Pvt. Ltd., 2023). We hope this summary addresses your concerns regarding the bioinformatics methods used in our gut microbiota analysis. In future studies, we will consider providing more extensive details if necessary, while maintaining a focus on the primary research objectives.

**13. The description of the histological scoring system is vague. It would be beneficial to provide more detailed criteria for the scoring of hepatic and colonic tissue damage, as well as examples or references to standard protocols.**

**Response:** Thank you for your insightful comment regarding the histological scoring system in our manuscript. To assess gut integrity, we utilized both macroscopic and microscopic evaluations. The macroscopic assessment followed a standard scoring system based on [4] which included parameters such as the presence of diarrhea, the severity of adhesions between the colon and adjacent organs, and the measurement of the large intestine's length along with inflammatory ulcerations. For microscopic examination, we adhered to protocols established by Chompre et al., 2022 [5], evaluating parameters like mucosal architectural disruption, muscular layer thickness, cellular infiltration, crypt abscess formation, and goblet cell depletion. These criteria were chosen due to their relevance in highlighting the detrimental effects of HFD on colonic health, as evidenced by increased damage scores in the HFD group compared to the LP and ND groups. This aligns with existing literature that demonstrates how high-fat diets can provoke inflammatory responses and structural changes in the colon [6,7]. Furthermore, we observed a reduction in colon length in the HFD group, consistent with previous studies [8,9], and noted structural changes that compromise colonic integrity and increase vulnerability to damage [10,11]. We hope this clarification addresses your concerns regarding the scoring criteria for histological assessments.

In case of hepatic scoring standard protocol with minor modifications was followed including parameters like- steatosis, immune infiltration, and hepatocyte ballooning [12]. Necessary data regarding macroscopic scoring, microscopic scoring of colonic integrity as well as, hepatic scoring is added in the S4 Table, S5 Table, and S3 Table respectively for better understanding.

**14. Many grammatical errors in the text throughout the manuscript were understandable. Thus, the language of the paper should be checked carefully again.**

**Response:** Authors are thankful for your suggestion. Necessary amends are made in the manuscript.

**Reviewer #1: This study investigates the potential of *Lactobacillus plantarum* KAD supplementation to mitigate metabolic disorders, particularly in the context of high-fat diet (HFD)-induced insulin resistance (IR) and associated hepatic injuries in mice. The manuscript has several promising aspects, but it requires revisions as several points need to be carefully revised before the next resubmission as follows:**

**1) Clarify the hypothesis of the study at the end of the Introduction section.**

**Response:** Thank you for your suggestion. Hypothesis is added at the end of the introduction section in the manuscript.

**2) How did you calculate the sample size for the animals included in this study?**

**Response:** Thank you for your questions regarding our study's methodology. For the sample size, we included six animals per group, which is statistically applicable in most animal studies, as supported by guidelines suggesting that sample sizes of this magnitude can provide sufficient power to detect significant differences [13,14].

**3) What is dose of L. plantarum per kg diet?**

**Response:** Thank you for raising this query but, we administered 10^10^ CFU of *L. plantarum* KAD orally per day per animal. Since we did not supplement the bacterial strain directly into the diet, calculating the dose per kg of diet was not feasible.

**4) The authors had to justify the selection of L. plantarum dose, supported by appropriate reference.**

**Response:** Authors thank the reviewer for his/her suggestion. Appropriate reference is added in section 2.2 regarding the dosage of *L. plantarum* KAD.

**5) In the Results section, remove the data from the text as it is already displayed in Figures.**

**Response:** Authors are thankful to the reviewer for suggesting this. All the necessary changes are made according to your suggestion.

**6) The formulation or nutrient composition of the experimental diet should be given.**

**Response:** Thank you for your valuable suggestion. Nutrient compositions of the experimental diets are provided in the S1 Table.

**7) English editing of the manuscript is highly recommended.**

**Response:** Authors are thankful for your recommendation. Language editing are made in the manuscript accordingly.

**References:**

1.	Cintra DE, Pauli JR, Araújo EP, Moraes JC, de Souza CT, Milanski M, et al. Interleukin-10 is a protective factor against diet-induced insulin resistance in liver. J Hepatol. 2008;48: 628–637. doi:10.1016/j.jhep.2007.12.017

2.	Pradhan AD. C-Reactive Protein, Interleukin 6, and Risk of Developing Type 2 Diabetes Mellitus. JAMA. 2001;286: 327. doi:10.1001/jama.286.3.327

3.	Barbier L, Ferhat M, Salamé E, Robin A, Herbelin A, Gombert J-M, et al. Interleukin-1 Family Cytokines: Keystones in Liver Inflammatory Diseases. Front Immunol. 2019;10. doi:10.3389/fimmu.2019.02014

4.	Appleyard CB, Wallace JL. Reactivation of hapten-induced colitis and its prevention by anti-inflammatory drugs. American Journal of Physiology-Gastrointestinal and Liver Physiology. 1995;269: G119–G125. doi:10.1152/ajpgi.1995.269.1.G119

5.	Chompre G, Sambolin L, Cruz ML, Sanchez R, Rodriguez Y, Rodríguez-Santiago RE, et al. A one month high fat diet disrupts the gut microbiome and integrity of the colon inducing adiposity and behavioral despair in male Sprague Dawley rats. Heliyon. 2022;8: e11194. doi:10.1016/j.heliyon.2022.e11194

6.	Cani PD, Neyrinck AM, Fava F, Knauf C, Burcelin RG, Tuohy KM, et al. Selective increases of bifidobacteria in gut microflora improve high-fat-diet-induced diabetes in mice through a mechanism associated with endotoxaemia. Diabetologia. 2007;50: 2374–2383. doi:10.1007/s00125-007-0791-0

7.	Wit M, Trujillo‐Viera J, Strohmeyer A, Klingenspor M, Hankir M, Sumara G. When fat meets the gut—focus on intestinal lipid handling in metabolic health and disease. EMBO Mol Med. 2022;14. doi:10.15252/emmm.202114742

8.	Hill AA, Kim M, Zegarra-Ruiz DF, Chang L-C, Norwood K, Assié A, et al. Acute high-fat diet impairs macrophage-supported intestinal damage resolution. JCI Insight. 2023;8. doi:10.1172/jci.insight.164489

9.	Papoutsis D, da Rocha SDC, Herfindal AM, Bøhn SK, Carlsen H. A High-Fat Western Diet Attenuates Intestinal Changes in Mice with DSS-Induced Low-Grade Inflammation. J Nutr. 2022;152: 758–769. doi:10.1093/jn/nxab401

10.	Xie Y, Ding F, Di W, Lv Y, Xia F, Sheng Y, et al. Impact of a high‑fat diet on intestinal stem cells and epithelial barrier function in middle‑aged female mice. Mol Med Rep. 2020. doi:10.3892/mmr.2020.10932

11.	Thomas S, Dilbarov N, Kelly J, Mercogliano G, Prendergast GC. Diet effects on colonic health influence the efficacy of Bin1 mAb immunotherapy for ulcerative colitis. Sci Rep. 2023;13: 11802. doi:10.1038/s41598-023-38830-2

12.	Kleiner DE, Brunt EM, Van Natta M, Behling C, Contos MJ, Cummings OW, et al. Design and validation of a histological scoring system for nonalcoholic fatty liver disease. Hepatology. 2005;41: 1313–1321. doi:10.1002/hep.20701

13.	Festing MFW, Altman DG. Guidelines for the Design and Statistical Analysis of Experiments Using Laboratory Animals. ILAR J. 2002;43: 244–258. doi:10.1093/ilar.43.4.244

14.	Dell RB, Holleran S, Ramakrishnan R. Sample Size Determination. ILAR J. 2002;43: 207–213. doi:10.1093/ilar.43.4.207
